# Principal component analysis and fine-tuned vision transformation integrating model explainability for breast cancer prediction

**DOI:** 10.1186/s42492-025-00186-x

**Published:** 2025-03-10

**Authors:** Huong Hoang Luong, Phuc Phan Hong, Dat Vo Minh, Thinh Nguyen Le Quang, Anh Dinh The, Nguyen Thai-Nghe, Hai Thanh Nguyen

**Affiliations:** 1 College of Information and Communication Technology, Can Tho University, Can Tho 900000, Vietnam; 2 Information Assurance Department, FPT University, Can Tho 900000, Vietnam; 3 Information Technology Department, FPT University, Can Tho 900000, Vietnam

**Keywords:** Vision Transformer, Multi-head locality large kernel self-attention, Principal component analysis

## Abstract

Breast cancer, which is the most commonly diagnosed cancers among women, is a notable health issues globally. Breast cancer is a result of abnormal cells in the breast tissue growing out of control. Histopathology, which refers to the detection and learning of tissue diseases, has appeared as a solution for breast cancer treatment as it plays a vital role in its diagnosis and classification. Thus, considerable research on histopathology in medical and computer science has been conducted to develop an effective method for breast cancer treatment. In this study, a vision Transformer (ViT) was employed to classify tumors into two classes, benign and malignant, in the Breast Cancer Histopathological Database (BreakHis). To enhance the model performance, we introduced the novel multi-head locality large kernel self-attention during fine-tuning, achieving an accuracy of 95.94% at 100× magnification, thereby improving the accuracy by 3.34% compared to a standard ViT (which uses multi-head self-attention). In addition, the application of principal component analysis for dimensionality reduction led to an accuracy improvement of 3.34%, highlighting its role in mitigating overfitting and reducing the computational complexity. In the final phase, SHapley Additive exPlanations, Local Interpretable Model-agnostic Explanations, and Gradient-weighted Class Activation Mapping were used for the interpretability and explainability of machine-learning models, aiding in understanding the feature importance and local explanations, and visualizing the model attention. In another experiment, ensemble learning with VGGIN further boosted the performance to 97.13% accuracy. Our approach exhibited a 0.98% to 17.13% improvement in accuracy compared with state-of-the-art methods, establishing a new benchmark for breast cancer histopathological image classification.

## Introduction

Breast cancer remains one of the most frequent and problematic cancers in women worldwide [1]. This malignancy begins in the breast tissue and can take different forms, including localized tumors and metastatic growths that spread to distant organs [2]. Its deadly nature originates from its ability to spread and invade other organs, significantly lowering survival rates. A histopathological study of breast tissue samples aided the diagnosis and staging off the tumor features [3]. Breast cancer causes serious health problems and can be fatal if not treated at an early stage. Thus, early detection and effective therapy are critical for combating this illness.

Breast cancer has a significant impact on both individuals and communities. The Global Cancer Observatory reports that female breast cancer accounted for 11.7% of all diagnoses (2.26 million cases), followed by lung cancer (2.21 million cases) and prostate cancer (1.41 million cases) in 2020 [1]. The incidence of breast cancer has increased over the past four decades [4]. Since the mid-2000s, the incidence of breast cancer among females has risen gradually, averaging approximately 0.5% per year [5]. Moreover, as reported in it was estimated that there would be 297,790 new cases of breast cancer and 43,170 deaths in the United States by 2023 [5]. In 2015, approximately 303,600 new cases of breast cancer and 70,400 fatalities were reported in China. The age-standardized incidence and death rates increased by 3.3% and 1.0%, respectively, between 2000 and 2015. These rates are expected to increase by more than 11% by the year 2030 [6]. There has been a notable increase in new cancer diagnoses in Republic of Korea, with 277,523 cases reported in 2021, marking an increase of 27,002 cases (10.8%) compared to 2020, and the number of reported cancer-related deaths in 2021 stands at 82,688 [7].

Breast cancer typically causes fear and death, which have an emotional impact on patients and their families. However, advances in medical research and computer science have resulted in various therapeutic options for the treatment of this disease. Surgery, chemotherapy, radiation therapy, hormonal therapy, targeted therapy, or a combination of these treatments may be used depending on the features of the tumor and the overall health of the patient. Furthermore, the interaction of artificial intelligence (AI) and medical research has inspired significant interest in employing computational techniques to improve illness diagnosis and treatment [8]. Deep learning showed great potential for evaluating medical imaging data such as mammograms, magnetic resonance imaging, and microscopic images for early diagnosis and categorization [9]. In addition, vision Transformer (ViT) models in deep learning have improved medical image analysis by better capturing spatial relationships and contextual information [10–12]. These developments have accelerated diagnostic procedures and contributed to individualized treatment planning by offering insights into tumor behavior and responses to therapy [13]. As AI research advances, collaborative efforts among computer scientists, medical professionals, and interdisciplinary teams can improve the precision and efficacy of breast cancer care, ultimately improving patient outcomes and reducing the burden of this devastating disease.

Previous research on categorizing breast cancer histopathology images has employed machine-learning algorithms such as convolutional neural networks (CNNs). Hameed et al. [14] presented an architecture using the fine-tuned VGG-16 and VGG-19 models in a CNN to identify breast cancer histopathology images with a high overall accuracy of 95.29%. However, methods related to visual explanations should be applied in the outcome to support the final decision. Furthermore, Mahraban Nejad et al. [15] suggested a VGG-19 pre-trained deep CNN (DCNN) model for extracting distinctive transferred features in the Breast Cancer Histopathological Database (BreaKHis) and achieved an average testing performance of 80.00% accuracy. Nevertheless, it was limited by the complexity and computational demands of VGG-19, and the study did not explore data augmentation or varying depth magnifications. Moreover, Saini and Susan [16] demonstrated the performance of VGGIN-Net using transfer learning and fine-tuning in the VGG-16 model. The proposed model achieved an average accuracy of 96.15% across all magnifications. However, VGGIN-Net recognizes the computational issues that VGG-based designs provide and strives to address them by changing the VGG-16 architecture.

Recent advances in deep learning techniques have enabled the creation of more sophisticated models that can capture detailed characteristics and patterns in histopathological images. Gupta and Bhavsar [17] coupled DenseNet for feature extraction with XGBoost for classification without relying on transfer learning and achieved an excellent average testing performance of 94.12%. However, this approach may require improvement in the transparency and interpretability of complicated models. In addition, Zhu et al. [18] used Inception and Squeeze-Excitation-Pruning for feature extraction, emphasizing staining, color transfer, and data augmentation. It exhibited an average testing performance of 83.73% at various magnification levels. However, the complexity of this method may cause issues with interpretation and practical use. Deniz et al. [19] also used fine-tuning in the AlexNet and VGG-16 models for feature extraction. This approach achieved a remarkable average accuracy of 91.05%, which can be further improved through data augmentation and preprocessing.

CNNs have long been the cornerstone of image classification tasks, including the classification of breast cancer histopathology images. Gour et al. [20] designed a model named ResHist for breast cancer histopathological image classification. The proposed model successfully classified breast cancer with an accuracy of 84.34%. In addition, this method yielded an accuracy of 92.52% when data augmentation was used. Using the FE-BkCapsNet model, Wang et al. [21] employed the advantages of CNN and CapsNet to classify the BreaKHis dataset and achieved impressive accuracy results (i.e., 40$\times$: 92.71%, 100$\times$: 94.52%, 200$\times$: 94.03%, and 400$\times$: 93.54%). Finally, Li et al. [22] proposed a CNN architecture for classifying benign and malignant breast cancer in histological images using DenseNet with the Squeeze-And-Excitation Network (SENet) module. This method achieved an average test accuracy of 87.9%. Moreover, several architectures were developed to adapt the requirements with more accuracy and lower cost, such as using ResNeX [23], DenseNet201 [24], and DarkNet53 [25]. However, the trust of these studies in only a single architectural family limits the exploration of potentially superior alternatives. It does not address the aspect of model interpretability, which is a significant concern in medical image analysis, where understanding the rationale behind the decisions of a model is paramount for building trust and ensuring its clinical relevance.

ViT has emerged as a promising alternative to traditional CNNs in the classification of breast cancer histopathological images. Shiri et al. [26] demonstrated that the SupCon-Vit model and principal component analysis (PCA) achieved an impressive result with an accuracy of 88.61%. However, the model still exhibits high performance with slightly low accuracy because it needs to configure the PCA correctly. Additionally, the Self-ViT-MIL method proposed in ref. [27] achieved an accuracy score of 91.47% and an area under the curve (AUC) of 0.9426. However, this study did not explain the outcome, although it demonstrated effectiveness in classifying histopathological images. A novel Deconv-Transformer (DecT) network model that incorporates color deconvolution in the convolutional layers was presented [28]. The research found that MaxViT had the best performance in image classification for breast cancer [29]. Both studies achieved average accuracies of 93.02% and 91.57% on the BreakHis dataset [29]. However, this study acknowledges that these models, particularly those with intricate attention schemes, such as CrossViT and SepViT, may require substantial computational resources and may not be adaptable to image data with varying resolutions and complexities.

PCA has been applied extensively in breast cancer diagnosis and classification using medical imaging data. Several studies have demonstrated the effectiveness of PCA in this domain. For example, Liu and Ma [30] used PCA to represent the information in region-of-interest images in a breast cancer recognition method based on support vector machines. Similarly, Hasan et al. [31] employed PCA for feature extraction in the development of breast cancer diagnostic models using various neural network architectures. Abdelsamee et al. [32] proposed a hybrid deep-learning approach that integrates CNN-based feature extraction with LR-PCA to address the multicollinearity problem among the deep features for breast lesion classification. Ragab et al. [33] utilized a framework based on multi-DCNNs with PCA-based feature selection for breast cancer classification. In addition, Sindhuja et al. [34] presented a breast cancer classification model that combines PCA and deep neural networks. These studies highlight the suitability and effectiveness of PCA as a preprocessing and feature extraction technique for breast cancer classification using medical imaging data.

Explanation techniques in machine learning provide insights into how models make predictions. These techniques enable users to gain deeper understanding and confidence in model outputs. For instance, Lu et al. [35] used Gradient-weighted Class Activation Mapping (Grad-CAM) after classifying brain tumors to explain the model decision and facilitate its future use by professionals and doctors. Furthermore, Grad-CAM can be applied for visual explanations of other diseases, such as breast cancer, chest cancer with COVID-19, and skin diseases [36–38]. In addition to human illnesses, Grad-CAM can also visualize crop diseases; refs. [39–41] demonstrated the effectiveness of visualization techniques in maize, bean, and coffee leaves, respectively. Local Interpretable Model-agnostic Explanations (LIME) and SHapley Additive exPlanations (SHAP) are techniques that provide several positive explanation results, which can help to visualize the model decision and create an easy method for doctors to determine the essential parts of the image to which to pay attention. For example, abnormal nasopharyngeal, lung, and breast positions have been applied to LIME and SHAP in various studies [42–44]. In addition, explaining the results builds greater confidence and enhances performance in classifying agricultural products. LIME and SHAP have been used to explain the predictions of the leaves of mulberries, potatoes, and tomatoes [45–47].

In summary, the current research on the histological classification of breast cancer shows promise for explanatory techniques. However, related machine learning works must be more accurate or distinguish between explanation techniques. Furthermore, data augmentation algorithms have been avoided, leading to data imbalance, which creates problems in increasing the accuracy of the model. Thus, addressing these research gaps requires the development of a new model that employs PCA as a preprocessing step for feature extraction from breast cancer histopathological images. In addition, there is a need to explore and propose new attention mechanisms that can effectively capture spatial and contextual information from histopathological images. A potential solution is the introduction of a new attention mechanism known as multi-head locality large kernel self-attention (MLLKSA). This mechanism combines the advantages of locality attention with large kernel layers, allowing the model to capture fine-grained details and broader spatial patterns within the histopathological images.

Overall, applying new technology such as ViT in computer vision helps researchers and developers to find different and effective means of creating new methods for image classification of breast cancer [48–50]. Thus, this study proposes a method that employs fine-tuning in ViT to classify microscopic images while using many technical tools to increase the accuracy, such as data augmentation and preprocessing. This study proposes an attention mechanism that combines locality self-attention and ample kernel attention. Furthermore, a visual explanation is provided for the final decision.

The contributions of this study are summarized as follows.

- An innovative approach using PCA is implemented to reduce the data dimensionality. This reduces the computational complexity while retaining crucial image features, mitigating overfitting, and enhancing the model accuracy.
- The impact of various data augmentation techniques (e.g., flipping, rotation, zooming, translation, brightness adjustment, and contrast adjustment) on the model accuracy is analyzed. Moreover, the utilization of combined augmentation methods assists the model precision and mitigates overfitting.
- The utilization of ViT architecture achieved a successful result in the average testing performance of all magnifications, with an accuracy of 96.65%. Thus, the experiment demonstrates the effectiveness of ViT architecture in classifying histopathological images.
- This study introduces the MLLKSA mechanism, which is a novel approach combining locality self-attention and large kernel attention. This fusion assists in increasing the model performance by drawing on the strengths of both attention mechanisms. The model demonstrates the efficacy of our proposed enhancement technique in various tasks through comprehensive experimentation and analysis.
- An integration of the SHAP, LIME, and Grad-CAM techniques is employed to increase the interpretability and reliability of deep learning models. The approach offers a clearer understanding of model predictions by explaining the decision-making process.

## Methods

### Methodology

#### Research implementation procedure

**Fig. 1:**
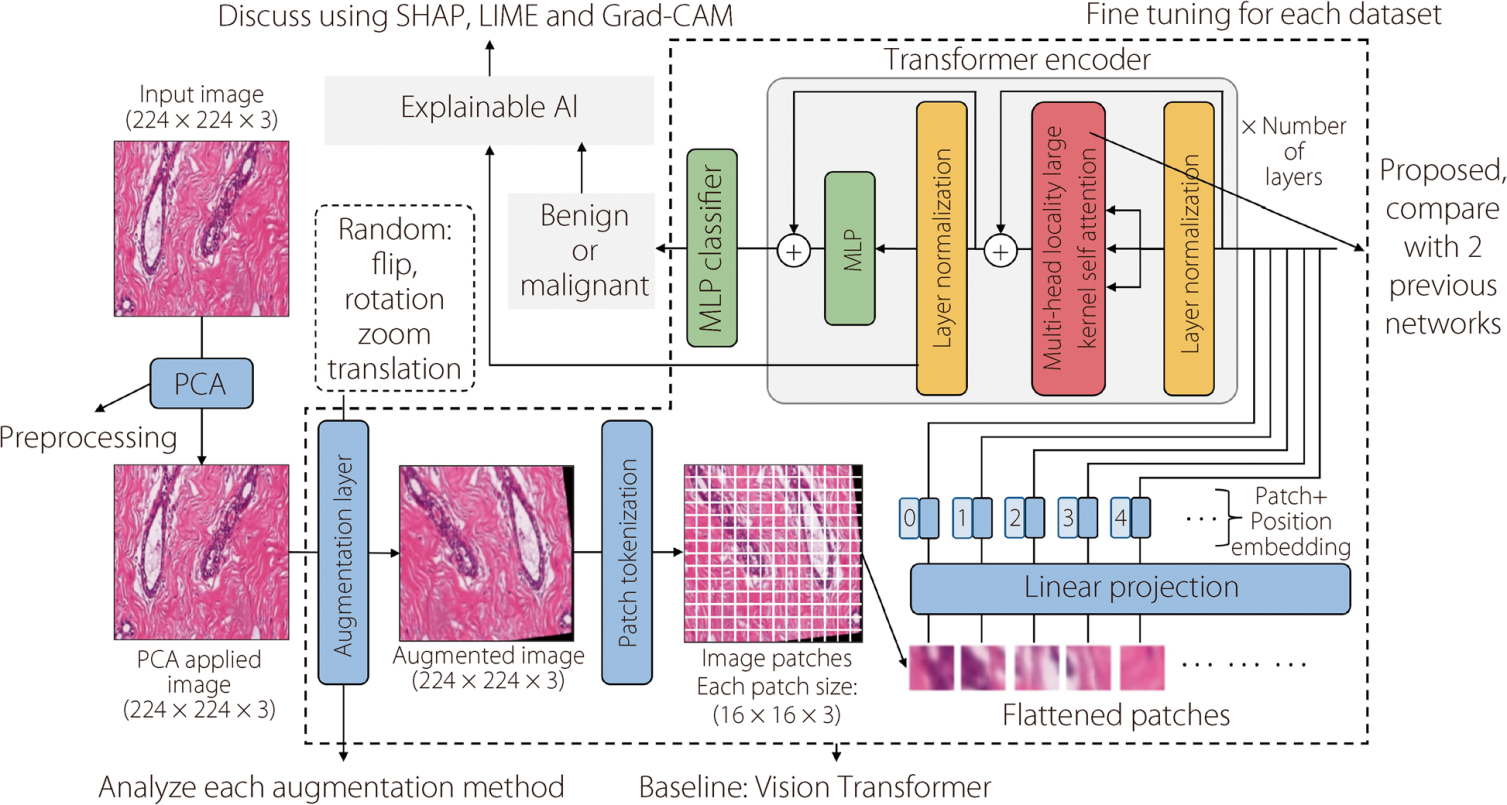
Implementation procedure flowchart

Figure 1 illustrates the procedure of this research. This can be described in eight steps as follows:

1. Input images: the input BreakHis dataset comprises over 9,000 microscopic images of breast tumor tissue obtained from 82 patients at various magnifications. The dataset is annotated with benign and malignant samples and maintained through collaboration with the P&D Laboratory in Brazil. Each image is standardized at 700 × 460 pixels, PNG format, and an 8-bit depth. This comprehensive dataset formed the foundation for the subsequent analysis and model development.
2. Data preprocessing: Data preprocessing techniques are employed to refine raw data for machin-learning tasks. PCA was selected as the dimensionality reduction technique owing to its key advantages. PCA can efficiently transform high-dimensional breast cancer histopathology images into a lower-dimensional space while preserving essential visual features. This reduction in dimensionality decreases the computational complexity and mitigates overfitting, which is a common challenge with limited medical image datasets. Importantly, the ability of PCA to capture informative data components aligns well with the BreakHis dataset, making it a well-suited preprocessing step for this breast cancer classification problem.
3. Data augmentation: Data augmentation is essential in expanding the size and diversity of the dataset used for training effective machine learning models. Techniques such as flipping, rotation, zooming, translation, brightness adjustment, and contrast adjustment are applied to create modified copies of the existing data. By applying these operations, the variability and robustness of the dataset are improved, contributing to better model generalization and performance.
4. Applying ViT with MLLKSA: the proposition of a ViT with MLLKSA represents a new sophisticated approach to feature extraction and classification. This method integrates locality self-attention and large kernel attention mechanisms. By exploiting these attention mechanisms, the model can capture the local and global dependencies inside the data to increase its ability to discern intricate patterns and structures.
5. Ensemble with VGGIN: The combination of ViT and VGGIN models increases the robustness and generalization capabilities of the overall system. By integrating diverse models, the ensemble approach mitigates individual model biases and errors, resulting in improved predictive performance and reliability. This collaborative framework enables comprehensive analysis and interpretation of the results, facilitating informed decision-making and model refinement.
6. Explaining the results with Grad-CAM, LIME, and SHAP: Using Grad-CAM, LIME, and SHAP for feature explanation and result validation underscores the commitment to transparency and interpretability in the model development. These methods provide insightful explanations for model predictions, offering valuable insights into the underlying decision-making processes. By applying these techniques, the research ensures that the model outcomes are comprehensible and trustworthy, fostering trust and confidence in the proposed approach.
7. Comparison of the proposed method with state-of-the-art approaches: A comparative analysis with state-of-the-art approaches assists in evaluating the efficacy and superiority of the proposed methodology. Through a comparison with established methodologies, the proposed method exhibits its innovation and advancement in the field, highlighting its contributions and potential impact. The research demonstrates the findings through careful comparisons and evaluations and establishes its relevance within the larger scientific community.
8. Presenting the results: The presentation of the results through meticulously organized tables and graphs increases the clarity and accessibility of the findings. The research facilitates straightforward interpretation and comprehension by structuring the results coherently and in a visually engaging manner.

#### Dataset and environment

The BreakHis [51] dataset includes 9,109 microscopic images of breast tumor tissue captured from 82 patients at various magnifications (40×, 100×, 200×, and 400×). Table 1 includes 2,480 benign and 5,429 malignant samples, providing a valuable resource for breast cancer research and diagnosis. An image is 700 × 460 pixels in PNG format, with three RGB channels and eight bits of depth per pixel. This dataset was developed using the P&D Laboratory - Pathological Anatomy and Cytopathology in Paraná, Brazil. The BreakHis dataset was divided into three subsets of 70%, 20%, and 10% for training, validation, and testing, respectively. This division ensures a balanced data distribution for model development, tuning, and evaluation.

**Table 1:** Number of images from BreakHis dataset

Main category		Benign	Malignant	Total
The number of images at each magnification level	40×	625	1370	1995
	100×	644	1437	2081
	200×	623	1390	2013
	400×	588	1232	1820
Total		2480	5429	7909

After dividing the dataset, random oversampling was applied to the training set to address the class imbalance issue. This technique duplicates instances of the minority class (benign) to balance the dataset, thereby ensuring that the model learns equally from both classes during training. Oversampling was applied only to the training set, leaving the validation and test sets unchanged, and preserving the integrity of the model evaluation.

To classify breast cancer using histopathological images, a testing environment was established with a focus on the use of essential libraries and frameworks. The environmental requirements encompassed Numpy, Pandas, Scikit-learn, OpenCV2, TensorFlow, and Keras, ensuring comprehensive support for data manipulation, machine learning algorithms, image processing, and deep learning model development. The Adam optimizer was used in the training setting with sparse categorical accuracy as the evaluation measure and sparse categorical cross-entropy as the loss function. Notably, all computations were run on a Kaggle P100 GPU to speed up the computations and allow for large-scale experimentation, ensuring efficient resource utilization and faster model training and evaluation.

For our study, we primarily used images at the 100× magnification level from the BreakHis dataset. All experiments were conducted using only 100× images, except for those in Scenario 4 (see Scenario 4: Results of combining all proposed methods section), in which images from all four magnification levels (40×, 100×, 200×, and 400×) were used to assess the impact of varying the magnification levels on the classification performance.

#### Data preprocessing

**Fig. 2:**
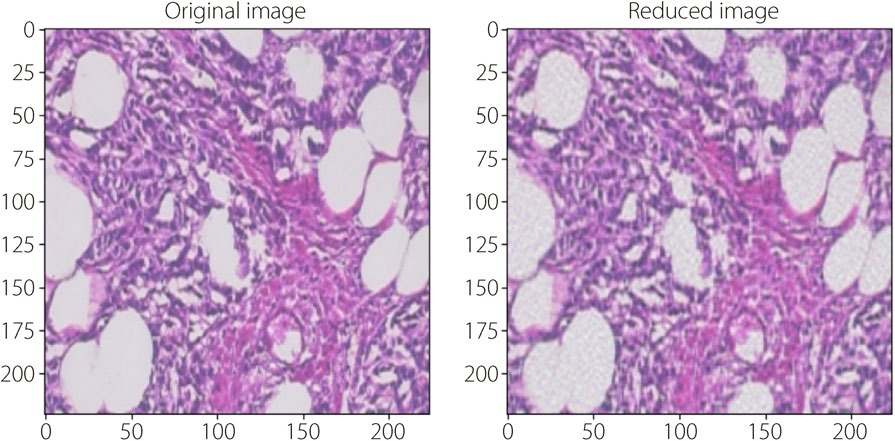
Effects of PCA dimensionality reduction on images from the BreakHis dataset

Data preprocessing techniques are essential for refining raw image data to enhance the performance of machine learning models. This subsection outlines the normalization and PCA processes applied to the breast cancer histopathological images. To classify breast cancer images, PCA can be applied to preprocess the image data effectively, as shown in Fig. 2. This figure illustrates the impact of applying PCA to breast cancer histopathological images. The left panel shows the original high-dimensional histopathology image, which contains various intricate tissue structures and patterns that are essential for diagnostic analysis. The right panel shows the same image after PCA-based dimensionality reduction. Despite reducing the data complexity, PCA effectively retains crucial visual features, ensuring that essential diagnostic information is preserved. This transformation not only reduces the computational requirements, but also mitigates the risk of overfitting in machine learning models, making PCA a valuable preprocessing step for breast cancer classification tasks.

The raw image data were first normalized to a range of 0 to 1 by dividing each pixel value by 255. This normalization step ensured that all pixel values lay within a consistent range, facilitating more stable and efficient subsequent processing. Mathematically, normalization can be represented as shown in Eq. 1:

1 $$\begin{aligned} X_{ij}^{\prime } = \frac{X_{ij}}{255} \end{aligned}$$

Consider an image represented by the matrix *X* with pixel values $X_{ij}$, where *i* represents the row index and *j* represents the column index. Moreover, $X_{ij}^{\prime }$ represents the normalized pixel value and 255 is the maximum pixel value in the image (i.e., assuming that the image is represented in the range of 0 to 255). This equation scales each pixel value by the maximum possible value, ensuring that all pixel values fall within the range of 0 to 1.

Following normalization, the image is segmented into color channels, typically referred to as red, green, and blue. Consequently, the image can be mathematically represented as a matrix $X^{(c)} \in \mathbb {R}^{m \times n}$, where *m* and *n* denote the image dimensions. PCA is then independently applied to each color channel $X^{(c)}$ to extract the principal components. The PCA algorithm computes the covariance matrix $C^{(c)} \in \mathbb {R}^{n \times n}$ of the color channel data, where *n* is the number of pixels in the channel. The covariance matrix is given by Eq. 2:

2 $$\begin{aligned} C^{(c)} = \frac{1}{n}\left(X^{(c)} - \bar{X}^{(c)}\right)^T\left(X^{(c)} - \bar{X}^{(c)}\right) \end{aligned}$$

In Eq. 2, $\bar{X}^{(c)}$ is the mean of the pixel values in channel *c*. Furthermore, PCA calculates the eigenvectors and eigenvalues of the covariance matrix $C^{(c)}$. The eigenvectors represent the principal components of the data and the eigenvalues indicate the variance captured by each principal component.

Let $U^{(c)}$ be the matrix of eigenvectors corresponding to the covariance matrix $C^{(c)}$ (from Eq. 2), sorted in descending order of eigenvalues. The transformed data $Z^{(c)}$ are obtained by projecting the original data onto the subspace spanned by the principal components, as shown in Eq. 3.

3 $$\begin{aligned} Z^{(c)} = X^{(c)}U^{(c)} \end{aligned}$$

Following PCA transformation, the data are denormalized by multiplying each pixel value by 255 to restore them to the original scale. Finally, the processed color channels are merged back into a single image, resulting in a preprocessed image that undergoes PCA-based dimensionality reduction and feature extraction. This preprocessing prepares the breast cancer histopathological images for classification tasks by reducing the data dimensionality while retaining important information. Thus, the computational complexity of the subsequent classification algorithms is reduced, while potentially increasing the performance by focusing on the most informative features extracted from the images.

#### Data augmentation

**Fig. 3:**
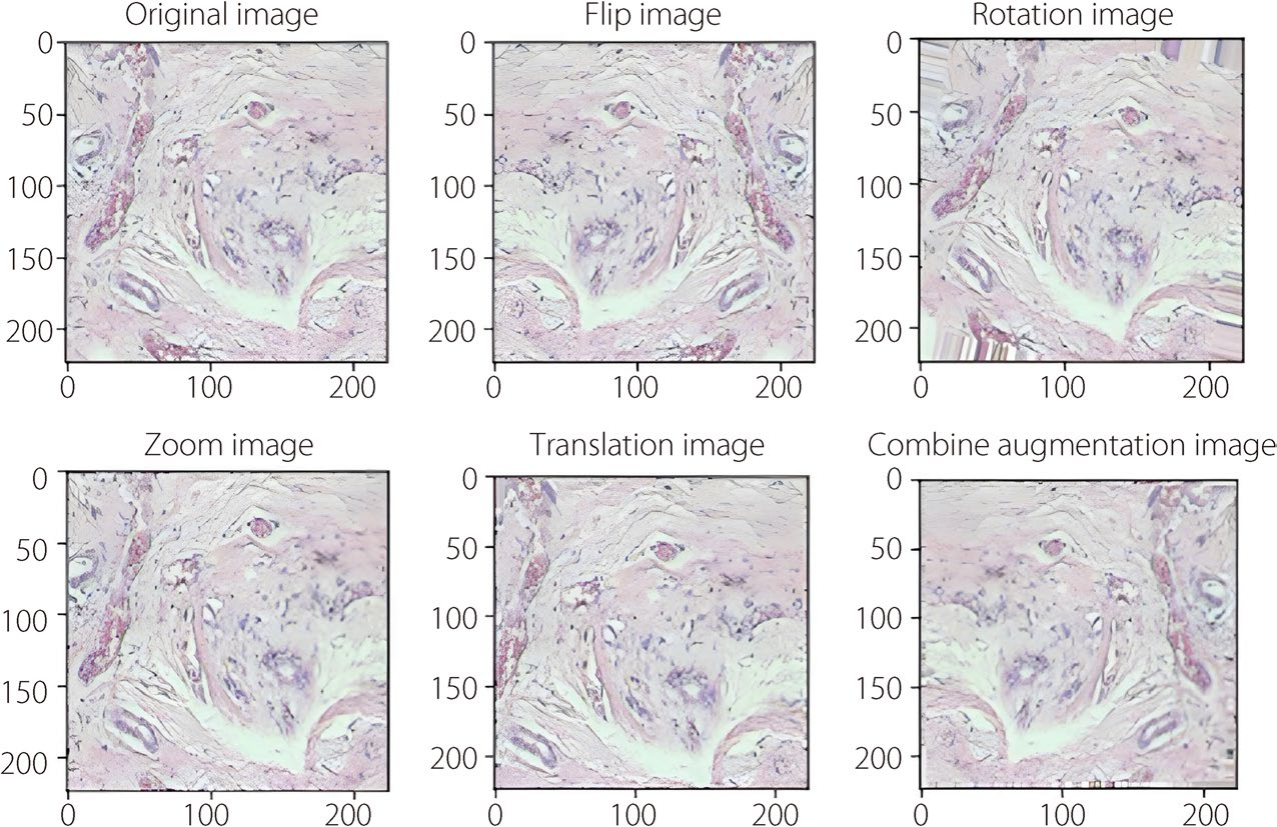
Visualization of the effect of data augmentation

Data augmentation involves the generation of new training data by applying various transformations to existing images. The model becomes more accurate by augmenting the dataset, and can better generalize to unseen data. Thus, data augmentation techniques such as flipping, rotation, zooming, translation, brightness adjustment, and contrast adjustment, as shown in Fig. 3, are widely used to improve the performance of classification models.

Image flipping is a fundamental method used for data augmentation. Flipping an image horizontally or vertically does not change its underlying features but provides additional variations for the model to learn from. Mathematically, flipping can be represented by the following transformations:

4 $$\begin{aligned} f(x, y) = (W - x, y) \end{aligned}$$

5 $$\begin{aligned} f(x, y) = (x, H - y) \end{aligned}$$

In the transformation equations for horizontal (Eq. 4) and vertical (Eq. 5) flipping, *x* and *y* represent the coordinates of the original image, respectively. The variable *W* denotes the image width. Similarly, *H* denotes the image height. Rotation is another important augmentation technique that introduces variations into the image orientation. The model becomes more invariant to rotational changes when the images are rotated to a certain degree. The rotation transformation can be represented using rotation matrices, as follows:

6 $$\begin{aligned} \left( \begin{array}{c} x' \\ y'\end{array}\right) = \left( \begin{array}{cc} \cos (\theta ) & -\sin (\theta ) \\ sin(\theta ) & \cos (\theta ) \end{array}\right) \left( \begin{array}{c}x\\ y\end{array}\right) \end{aligned}$$

The transformation (Eq. 6) illustrates the rotation of an image by angle $\theta$. In this equation, (*x*, *y*) are the coordinates of a pixel in the original image and (*x*’, *y*’) are the coordinates of the corresponding pixel in the rotated image. The matrix in Eq. 6 represents the rotation matrix that performs the rotation operation. The cosine and sine functions determine the extent to which the pixel is displaced in the *x*- and *y*-directions, respectively, based on the angle $\theta$. The pixel coordinates in the rotated image are obtained by multiplying the original pixel coordinates by the rotation matrix.

Zooming and translation are techniques that alter the scale and position of images, respectively. Zooming changes the size of the objects in the image by scaling, as described in Eq. 7, whereas translation shifts the objects within the image by moving them to different positions, as shown in Eq. 8. These transformations can be represented as

7 $$\begin{aligned} f(x, y) = (x \times s_x, y \times s_y) \end{aligned}$$

8 $$\begin{aligned} f(x, y) = (x + t_x, y + t_y) \end{aligned}$$

In Eq. 7, $s_x$ and $s_y$ are the scaling factors for the width and height, respectively. In Eq. 8, $t_x$ and $t_y$ represent the translation offsets along the *x*- and *y*-axes, respectively.

Brightness and contrast adjustments modify the intensity distribution of images. By changing the brightness and contrast levels, the images become more vigorous with variations in the lighting conditions. These adjustments can be represented using simple arithmetic operations, as follows:

9 $$\begin{aligned} f(x) = x + \Delta b \end{aligned}$$

10 $$\begin{aligned} f(x) = \alpha \times x + \beta \end{aligned}$$

In Eq. 9, the brightness adjustment involves shifting the intensity values of all pixels by a fixed number $\Delta b$, which can either lighten or darken the entire image uniformly. Furthermore, contrast adjustment, as described by Eq. 10, changes the distribution of intensity values across the image. By scaling the intensity *x* of each pixel by a factor $\alpha$ and then adding an offset $\beta$, the contrast between different image parts can be intensified or softened.

In summary, data augmentation techniques are necessary to improve the robustness and generalizability of breast cancer histopathological image classification models. Combining these techniques helps the model to learn more diverse features, leading to better performance in real-world scenarios.

#### Fine-tuning of ViT model

ViT has become a well-known architecture in computer vision, representing a paradigm shift from CNN to Transformer-based models. Unlike traditional CNNs that rely on convolutional layers for feature extraction, ViT uses self-attention mechanisms to capture global dependencies inside an image. This enables ViT to process images effectively as sequences of patches, thereby overcoming some of the limitations of CNNs in handling long-range dependencies. Fine-tuning is essential for adapting pre-trained ViT models to specific downstream tasks. The model learns task-specific features by fine-tuning and improving its performance on the target task. Fine-tuning involves updating the weights of the pre-trained ViT model using task-specific data while retaining the knowledge gained from the pre-training phase.

**Figure:**
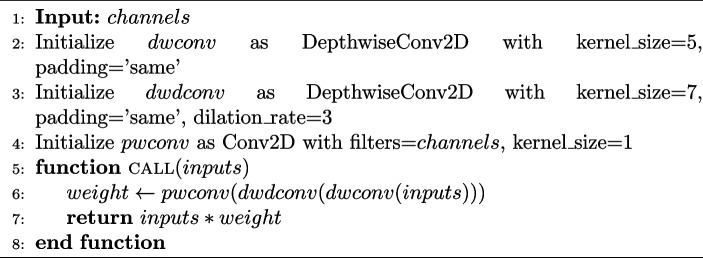
**Algorithm 1** Large kernel layer

**Fig. 4:**
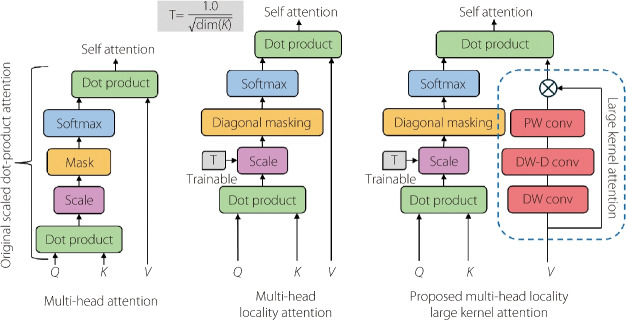
Comparison of self-attention among multi-head attention, multi-head locality attention, and proposed MLLKSA

Employing the recent advancements in this field, Fig. 4 shows the introduction of a novel attention mechanism known as MLLKSA for fine-tuning ViT models. MLLKSA combines the strengths of multi-head locality attention and a large kernel layer to increase the ability to capture both local and global contexts in an image. The large kernel layer component of MLLKSA employs several convolutional operations to capture contextual information across different receptive fields. Specifically, it initializes three convolutional layers, as shown in Algorithm 1: depthwise convolution (dwconv) with a kernel size of 5 and ‘same’ padding, depthwise dilated convolution (dwdconv) with a kernel size of 7, ‘same’ padding, and a dilation rate of 3, and pointwise convolution (pwconv) with a kernel size of 1. These convolutional layers enable the model to learn hierarchical representations at multiple scales, thereby facilitating more efficient feature extraction.

**Figure:**
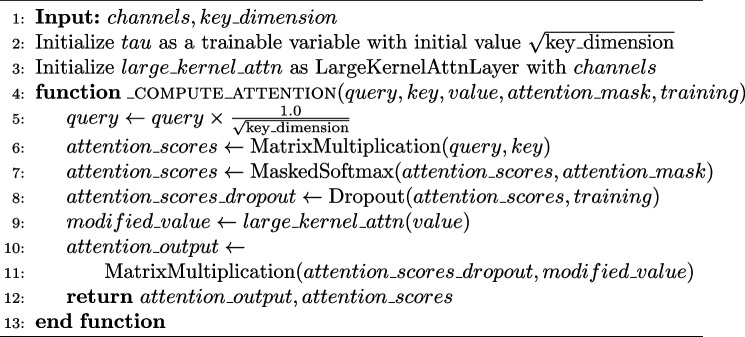
**Algorithm 2** Multi-head locality self-attention with large kernel layer

By integrating MLLKSA into the fine-tuning process of ViT models in Algorithm 2, we aim to enhance the model performance across various computer vision tasks. The proposed attention mechanism offers a promising avenue for improving the adaptability and generalization capabilities of ViT models, potentially leading to state-of-the-art performance in diverse real-world applications without excessive computational overheads.

#### SHAP, LIME, and Grad-CAM

SHAP, LIME, and Grad-CAM are essential tools in computer science, particularly for interpreting complex models such as the CNN and ViT. SHAP offers insights into feature importance at both the global and local levels, enhancing the understanding of the model predictions. LIME aids in increasing the transparency and reliability of image classification tasks by providing human-understandable explanations for complex model predictions. Grad-CAM is a valuable technique for interpreting decisions in histopathological image classification, allowing researchers and clinicians to identify the most significant regions contributing to classification decisions.

To illustrate, SHAP is grounded in cooperative game theory, specifically in the concept of Shapley values, which allocate credit to each feature based on its contribution to the prediction. Furthermore, SHAP quantifies the importance of individual pixels or image regions in influencing the decision of a model. Mathematically, the Shapley value for feature $\phi _i$ is computed as the average marginal contribution of that feature across all possible feature subsets, as shown in Eq. 11.

11 $$\begin{aligned} \phi _i = \sum _{S \subseteq N \setminus \{i\}} \frac{|N|!}{|S|!(|N|-|S|-1)!} (f(S \cup \{i\}) - f(S)) \end{aligned}$$

**Fig. 5:**
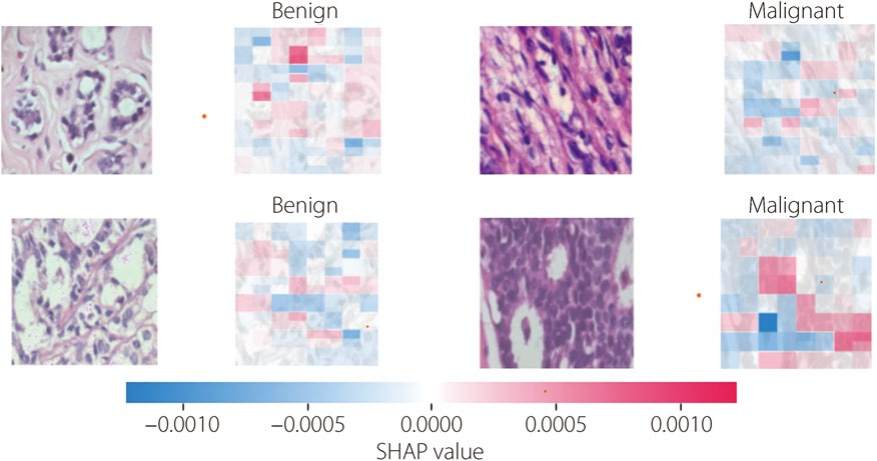
Results of classifying histopathological images after applying SHAP

In Eq. 11, *N* represents the set of all features, *f*(*S*) denotes the model prediction when considering only the features in subset *S*, and |*S*| denotes the cardinality of subset *S*. By computing these Shapley values for each feature, SHAP comprehensively explains the decision-making process of the model, revealing the relative importance of different image regions in driving the classification outcome. By visualizing the SHAP values as heatmaps overlaid on the original images, as shown in Fig. 5, researchers can gain actionable insight into the histopathological features that are indicative of malignancy or specific cancer subtypes. This visualization enables the identification of regions of interest within the images, facilitating qualitative and quantitative analyses of the underlying pathological characteristics.

In addition, LIME is a powerful tool in this domain, assisting in the interpretability of complex machine learning models for image classification tasks. LIME is essential for increasing the transparency and reliability of such models by providing human-understandable explanations of their predictions. Mathematically, as shown in Eq. 12, let *f* represent the complex black-box model under consideration and *x* denote the input image. LIME seeks to approximate the behavior of *f* around *x* using an interpretable model *g*. This approximation is achieved by solving the following optimization problem to determine the optimal $g^*$:

12 $$\begin{aligned} g^* = \arg \underset{g \in G}{\min }\ \mathcal {L}(f, g, \pi _x) + \Omega (g) \end{aligned}$$

In this equation, $g^*$ is the optimal interpretable model that best approximates the behavior of *f* near instance *x*. Set *G* represents all possible interpretable models from which *g* is selected and includes models that are simple and easy to understand, such as linear models or decision trees. The loss function $\mathcal {L}(f, g, \pi _x)$ measures the discrepancy between the predictions of the black-box model *f* and interpretable model *g*, weighted by the proximity measure $\pi _x$. The proximity measure $\pi _x$ assigns weights to samples based on their distance from *x*, assigning higher importance to samples closer to *x*. The term $\Omega (g)$ is a regularization term that is used to control the complexity of the interpretable model *g*, which acts as a penalty to prevent overfitting and encourages simplicity. By optimizing over the set *G*, LIME aims to find the interpretable model $g^*$ that closely approximates the behavior of *f* around input instance *x*.

**Fig. 6:**
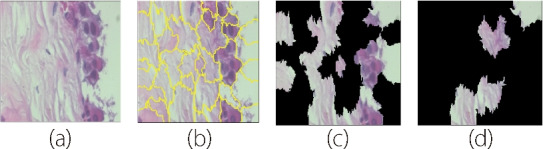
Description of LIME: **a** Original image; **b** Generating superpixels using the quickshift segmentation algorithm; **c** Generating image perturbations by turning some superpixels in the image on and off; and **d** Displaying LIME explanation (image with top features) after proceeding through the calculation steps

As shown in Fig. 6, LIME aids in identifying critical histological features that contribute to the classification of malignant and benign tissues. These features may include tumor cells, architectural patterns, nuclear morphology, and stromal characteristics. Moreover, LIME empowers clinicians and researchers to validate and refine the underlying diagnostic criteria to improve the accuracy and reliability of breast cancer diagnoses. In addition, the interpretability offered by LIME fosters greater transparency in the decision-making process for integrating machine-learning algorithms into clinical practice while ensuring accountability and trustworthiness.

Despite its usefulness in providing local explanations for complex computer vision models, LIME has several limitations, including its reliance on the fidelity of local approximations, sensitivity to perturbation strategies and the neighborhood size, assumptions of model consistency, and a trade-off between interpretability and global understanding. These constraints highlight the need for cautious interpretation and supplementation with other interpretability techniques, particularly in critical domains such as medical diagnosis.

The usefulness of Grad-CAM in classifying breast cancer histopathological images is based on its ability to provide visual explanations for the CNN. Traditional CNNs require greater interpretability to make it easier to discern the features to be prioritized when making predictions. Grad-CAM addresses this limitation by generating heat maps that highlight the most influential regions inside an image in the classification process.

**Fig. 7:**
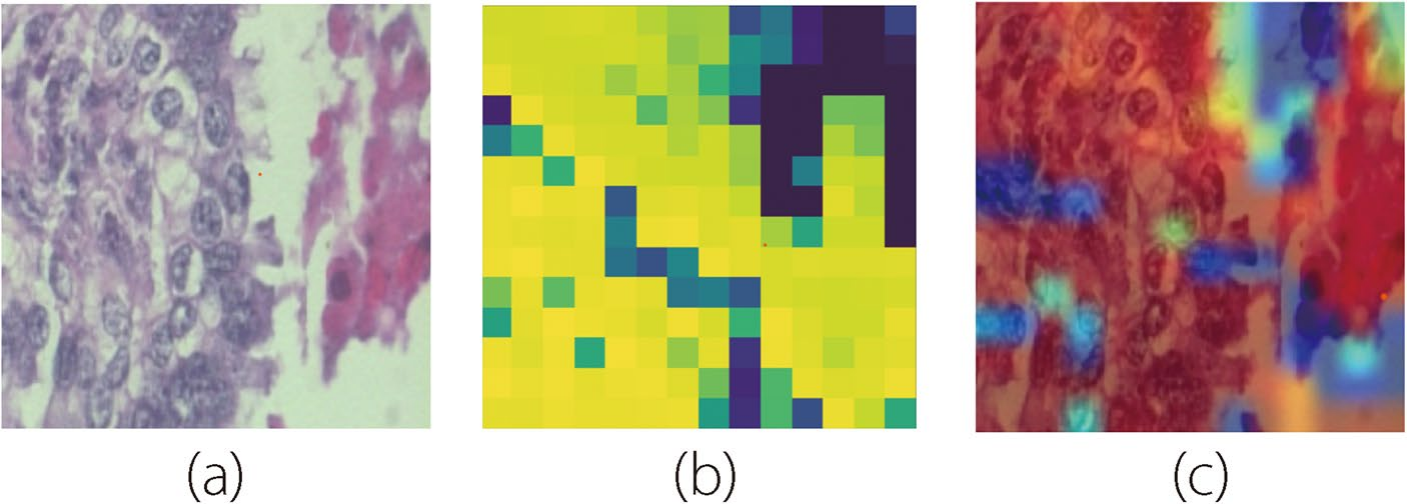
Grad-CAM applies a heatmap in a histopathological image: **a** Original image; **b** Displaying the heatmap of the original image; and **c** Displaying the original image with its heatmap

By overlaying these heatmaps onto the original image, as shown in Fig. 7, researchers gain insights into the histopathological features that drive the model decisions, facilitating a deeper understanding of the underlying biological mechanisms associated with different cancer types. Mathematically, Grad-CAM computes the gradient of the target class score based on feature maps of the last convolutional layer. Let $A^k$ (Eq. 13) represent the activation map of the $k\_th$ convolutional layer and $a_k^c$ (Eq. 13) indicate the importance of the $k\_th$ feature map for class *c*. The Grad-CAM heatmap for class *c* is computed as follows:

13 $$\begin{aligned} \text {Grad-CAM}^c = \text {ReLU} \left( \sum _k \alpha _k^c A^k \right) \end{aligned}$$

In Eq. 13, the rectified linear unit function ensures that only positive contributions are considered, highlighting the regions relevant to the target class. In summary, SHAP, LIME, and Grad-CAM are valuable tools for interpreting breast cancer histopathological images. Its ability to generate visual explanations enhances the transparency and interpretability of deep learning models, allowing researchers and clinicians to make more informed decisions regarding diagnoses and patient care. Finally, the comparison of the Grad-CAM, LIME, and SHAP techniques in Table 2 provides insights into their respective strengths and characteristics for interpreting and explaining the model predictions. Because these techniques have different interpretability requirements and computational constraints, practitioners can select the most suitable approach based on their specific requirements.

**Table 2:** A comparison of Grad-CAM, LIME, and SHAP based on visualization, interpretability, explanation focus, computational efficiency, and consistency

	Grad-CAM	LIME	SHAP
Visualization	Heatmap over input image	Perturbed images with superpixel influence	Feature importance over input image or bar plot
Interpretability	Intuitive spatial relevance	Granular influence of image segments	Theoretically grounded feature importance
Explanation focus	Areas of image influencing the output	Influence of superpixel presence or absence	Contribution of individual features to the output
Computational efficiency	Moderate	Low (requires multiple evaluations)	Low (computationally intensive)
Consistency	Relatively consistent	Can vary due to randomness in perturbation	Consistent with proper sampling

### Experiments

#### Performance metrics

Performance metrics are essential for evaluating the effectiveness of machine learning models, particularly in medical image classification tasks such as breast cancer diagnosis. The accuracy, recall, precision, F1-score, receiver operating characteristic (ROC) curve, AUC, Matthews correlation coefficient (MCC), Cohen’s kappa, and critical success index (CSI) are commonly used metrics in this domain.

The accuracy (Eq. 14) is measured using true positives (TP), true negatives (TN), false positives (FP), and false negatives (FN). TP illustrates instances in which the model accurately identifies malignant tumors, whereas TN represents instances in which benign tumors are correctly identified as benign. Conversely, FP occurs when the model incorrectly classifies benign tumors as malignant, posing a potential risk of misdiagnosis. In contrast, FN highlights instances in which malignant tumors are mistakenly labeled as benign.

14 $$\begin{aligned} Accuracy = \frac{TP + TN}{TP + TN + FP + FN} \end{aligned}$$

The recall (Eq. 15), also known as the sensitivity, quantifies the ability of the model to identify positive instances from all actual positive instances correctly, and is expressed as

15 $$\begin{aligned} Recall = \frac{TP}{TP + FN} \end{aligned}$$

The precision (Eq. 16) calculates the proportion of correctly predicted positive instances out of all instances that are predicted as positive, and is defined as

16 $$\begin{aligned} Precision = \frac{TP}{TP + FP} \end{aligned}$$

The F1-score (Eq. 17) is the harmonic mean of the precision and recall, offering a balanced measure of the model performance:

17 $$\begin{aligned} F_1 = 2 \times \frac{Precision \times Recall}{Precision + Recall} \end{aligned}$$

The MCC (Eq. 18) provides a more balanced metric for binary classification, particularly in cases where the classes are imbalanced. It considers the TP, TN, FP, and FN and is expressed as

18 $$\begin{aligned} MCC = \frac{(TP \times TN) - (FP \times FN)}{\sqrt{(TP + FP)(TP + FN)(TN + FP)(TN + FN)}} \end{aligned}$$

Cohen’s kappa (Eq. 19) measures the level of agreement between two raters or classification systems while accounting for the possibility of agreement occurring by chance. It is calculated as

19 $$\begin{aligned} \kappa = \frac{p_o - p_e}{1 - p_e} \end{aligned},$$

where $p_o$ is the relative observed agreement among raters and $p_e$ is the hypothetical probability of chance agreement.

The CSI (Eq. 20) is another performance metric that is used to evaluate classification models and is particularly useful in situations where false negatives and false positives are of concern. It is expressed as

20 $$\begin{aligned} CSI = \frac{TP}{TP + FN + FP} \end{aligned}.$$

The ROC curve is a graphical representation of the performance of a model across different thresholds. It plots the TP rate (recall) against the FP rate (1 - specificity) at various threshold settings. The AUC quantifies the area under the ROC curve, reflecting the ability to distinguish between classes.

In addition, the learning rate determines the step size taken during optimization, and Adam used in this research requires a smaller learning rate (i.e., starting at 0.001). A higher learning rate may result in faster convergence; however, it risks overshooting the optimal solution. In comparison, a lower learning rate may lead to slower convergence but with potentially more stable results. In the training setup, the number of epochs was set to 200, with early stopping implemented using the patience of 15 epochs based on the validation loss. Early stopping is a technique that terminates training when a predefined criterion, such as the validation loss or accuracy, fails to improve over a certain number of epochs. By preventing the model from continuing to train on potentially overfitting data, early stopping helps to generalize unseen data better and improves the model efficiency. These performance metrics provide valuable insights into the strengths and weaknesses of a model, enabling researchers to optimize algorithms for accurate breast cancer histopathological image classification.

#### Scenario 1: Effect of PCA on preprocessing BreakHis

In Scenario 1 of applying ViT models, the effect of PCA on data preprocessing was investigated to discern its impact on the classification performance. A comparison of the results obtained with and without PCA preprocessing was conducted to analyze the efficacy of PCA. The comparative analysis presented in Table 3 highlights the notable improvements in various performance metrics with the inclusion of PCA. Specifically, when PCA was applied, there was a discernible enhancement across the precision, recall, F1-score, and accuracy metrics compared to the scenario without PCA preprocessing. As shown in Table 3, the study indicated that using PCA led to an increase in precision from 84.39% to 87.58%, recall from 87.59% to 90.00%, F1-score from 85.96% to 88.78%, and accuracy from 80.91% to 84.25%. These improvements emphasize the beneficial impact of PCA in preprocessing the input data, which creates better discrimination between different classes.

**Table 3:** Comparative results of the effects of PCA

Method	Precision	Recall	F1-score	Accuracy	MCC	Cohen’s kappa	CSI	Epochs
**No-PCA**	84.39%	87.59%	85.96%	80.91%	0.5623	0.5614	0.7538	15
**PCA**	87.58%	90.00%	88.78%	84.25%	0.6246	0.6239	0.7982	83

In addition, as shown in Table 3, applying PCA improved the MCC from 0.5623 to 0.6246, Cohen’s kappa from 0.5614 to 0.6239, and CSI from 0.7538 to 0.7982. These enhancements indicate a stronger correlation between the predicted and actual classes, a higher agreement beyond chance, and a better ratio of true positives to all relevant cases, respectively.

**Fig. 8:**
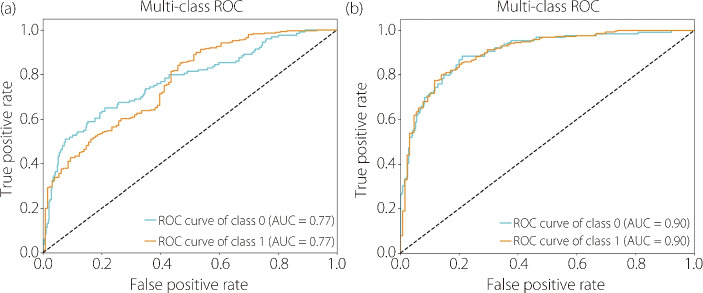
**a** ROC curve without PCA and **b** ROC curve with PCA

Moreover, the epochs differed with 15 for no-PCA and 83 for PCA because early stopping was used to prevent overfitting and improve the generalization performance. Furthermore, Fig. 8 shows that the ROC curve with PCA was higher than that without PCA (i.e., towards the upper-left corner of the plot), indicating a better discrimination ability as a higher true positive rate was achieved while a lower false positive rate was maintained across various threshold settings.

In summary, the results of this comparative analysis emphasize the significance of PCA as a preprocessing technique for improving the performance of ViT models. By effectively reducing the data dimensionality and preserving crucial information, PCA contributes to more discriminative representations, thereby elevating the classification accuracy and overall effectiveness of ViT models in various computer vision tasks.

#### Scenario 2: Effect of data augmentation techniques

Data augmentation is a crucial step that involves the generation of additional training images by applying transformations to the original dataset. These transformations aim to diversify the training data and improve the model generalization. Table 4 shows the effectiveness of the different data augmentation techniques in improving the classification accuracy. Each row in the table corresponds to a specific augmentation method and shows the resulting precision, recall, F1-score, accuracy, and number of epochs.

**Table 4:** Comparative results of the effects of data augmentation

Method	Precision	Recall	F1-score	Accuracy	MCC	Cohen’s kappa	CSI	Epochs
**None**	87.58%	90.00%	88.78%	84.25%	0.6246	0.6239	0.7982	83
**Flip**	89.77%	93.79%	91.74%	88.31%	0.7197	0.7177	0.8474	90
**Rotation**	93.49%	94.14%	93.81%	91.41%	0.7976	0.7975	0.8835	90
**Zoom**	92.33%	95.52%	93.90%	91.41%	0.7953	0.7940	0.8850	85
**Translation**	96.40%	92.41%	94.37%	92.36%	0.8271	0.8253	0.8933	70
**Brightness adjustment**	69.17%	92.07%	78.99%	66.11%	−0.0031	−0.0023	0.6528	139
**Contrast adjustment**	88.29%	62.41%	73.13%	68.26%	0.4046	0.3704	0.5764	16
**Combination (flip, rotation, zoom, translation)**	95.44%	93.79%	94.61%	92.60%	0.8286	0.8282	0.8977	119

The analysis showed notable improvements in accuracy with the incorporation of data augmentation techniques. For example, the ‘flip’ technique resulted in a significant increase in accuracy from 84.25% to 88.31% compared to the scenario with no data augmentation, representing a percentage increase of approximately 4.06%. Similarly, techniques such as ‘rotation’ and ‘zoom’ yielded substantial improvements in accuracy of 91.41%, representing percentage increases of approximately 7.16% and 7.16%, respectively.

Moreover, certain augmentation techniques resulted in superior performance in specific metrics. For instance, the ‘translation’ technique achieved the highest precision of 96.40%, showing its effectiveness in minimizing false positives. The “brightness adjustment” technique increased the recall, although it led to a significant decrease in the precision and F1-score, highlighting the importance of balanced augmentation strategies. Furthermore, the combination technique, which integrated multiple augmentation methods, including flipping, rotation, zooming, and translation, achieved a high accuracy of 92.60%, demonstrating the potential benefits of using diverse augmentations. In addition, the early stopping affected the epochs throughout the augmentation techniques. This helped the model to avoid continuing to learn from potentially noisy or overfitting data, thereby allowing it to generalize better to unseen examples.

In addition, as shown in Table 4, the data augmentation techniques significantly improved the MCC, Cohen’s kappa, and CSI. For example, the ‘flip’ method increased the MCC from 0.6246 (no augmentation) to 0.7197, Cohen’s kappa from 0.6239 to 0.7177, and CSI from 0.7982 to 0.8474. Techniques such as ‘rotation’ and ‘zoom’ further enhanced these metrics, with the MCC reaching up to 0.7976, Cohen’s kappa up to 0.7975, and CSI up to 0.8850. The combined augmentation method achieved the highest values, with an MCC of 0.8286, Cohen’s kappa of 0.8282, and CSI of 0.8977. These improvements indicate stronger correlations between the predicted and actual classes, higher agreement beyond chance, and a greater proportion of correct positive predictions, underscoring the effectiveness of data augmentation in enhancing the model reliability and performance.

**Fig. 9:**
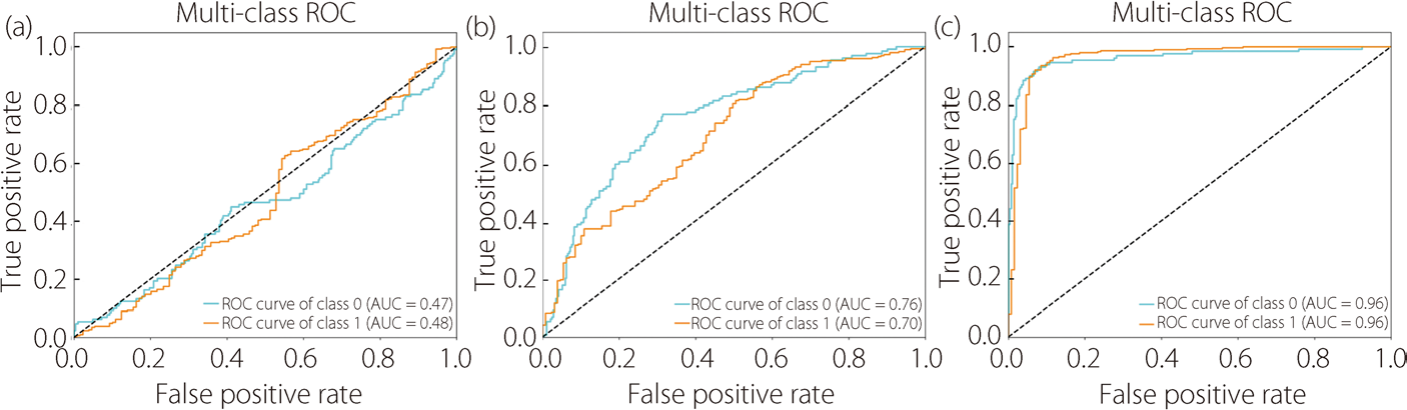
The ROC curve of **a** brightness adjustment, **b** contrast adjustment, and **c** combination

The ROC curve is shown in Fig. 9, which indicates that the combination methods were truly effective towards the upper-left corner of the plot. In addition, a comparison of the combined method with other augmentations showed that the AUC increase reached 0.96, with the highest increase of 0.49. In conclusion, the analysis highlighted the significant impact of data augmentation techniques on the classification performance of ViT models. By diversifying the training data, augmentation techniques contribute to improved generalization, robustness, and overall accuracy, facilitating more effective deployment of ViT models in real-world applications.

#### Scenario 3: Effect of attention mechanism

Scenario 3 involved evaluating the proposed MLLKSA. This attention mechanism introduces a novel approach for capturing dependencies within the input data. Table 5 compares the performance of the proposed MLLKSA with two existing attention mechanisms: multi-head self-attention and multi-head locality self-attention.

**Table 5:** Comparative results of the effects of the proposed self-attention

Method	Precision	Recall	F1-score	Accuracy	MCC	Cohen’s kappa	CSI	Epochs
**Multi-head self-attention**	95.44%	93.79%	94.61%	92.60%	0.8286	0.8282	0.8977	119
**Multi-head locality self-attention**	95.47%	94.48%	94.97%	93.08%	0.8388	0.8386	0.9043	132
**Multi-head locality large kernel self-attention**	97.56%	96.55%	97.05%	95.94%	0.9055	0.9054	0.9428	50

The analysis indicated a substantial improvement in accuracy with the adoption of the proposed MLLKSA mechanism. Compared with the baseline multi-head self-attention, the proposed mechanism achieved an accuracy of 95.94%, representing a significant increase of approximately 3.34%. Compared with multi-head locality self-attention, MLLKSA exhibited a dramatic accuracy improvement of approximately 2.86%. Moreover, the differences across epochs were manageable because of the early stopping technique.

**Fig. 10:**
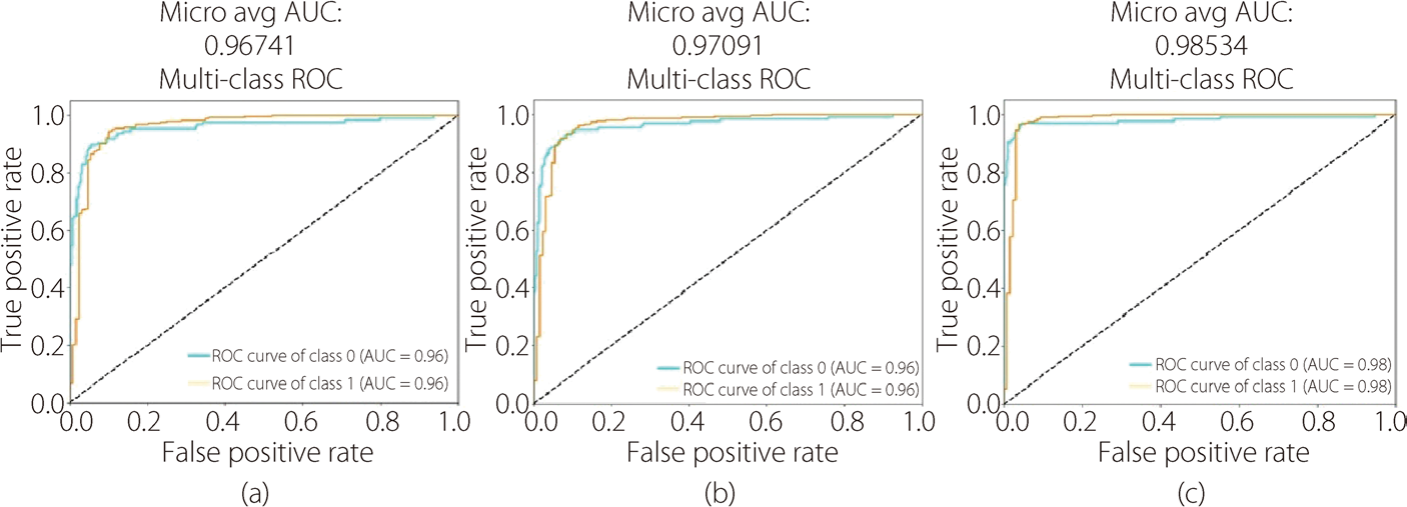
**a** Multi-head self-attention, **b** Multi-Head Locality Self-Attention, and **c** MLLKSA

Moreover, the proposed attention mechanism outperformed its counterparts in terms of the precision, recall, F1-score, MCC, Cohen’s kappa, and CSI metrics. In addition, the micro average AUC of the MLLKSA, as shown in Fig. 10, was higher than that of the other mechanisms and reached 0.98534.

These results highlight the effectiveness of the proposed MLLKSA mechanism in enhancing the discriminative power of ViT models. By incorporating the locality and significant kernel components, this attention mechanism enables the model to capture relevant features at multiple scales. Furthermore, the observed improvements underscore the potential of innovative attention mechanisms to advance the capabilities of ViT models.

#### Scenario 4: Results of combining all proposed methods

In Scenario 4, the focus was on evaluating the performance of the proposed method, which integrates various techniques and components to enhance the effectiveness of the ViT model. The proposed method encompasses preprocessing steps such as PCA, data augmentation, and feature extraction using MLLKSA.

Table 6 presents the performance of the proposed method across different magnifications during training and testing. The results demonstrate the promising performance of the proposed method at all magnifications. At the lowest magnification of 40×, the proposed model achieved an accuracy of 95.27%, which gradually improved to 98.09% at 400× magnification. This indicated the ability to generalize and adapt to varying magnification levels effectively during the training and testing phases. Notably, the proposed method achieved an impressive average testing performance of 96.65%.

**Table 6:** Results of the proposed method

Method	Training and testing magnification	Precision	Recall	F1-score	Accuracy	MCC	Cohen’s kappa	CSI
Proposed method	40×	96.39%	96.74%	96.56%	95.27%	0.8900	0.8899	0.9336
	100×	97.56%	96.55%	97.05%	95.94%	0.9055	0.9054	0.9428
	200×	98.56%	97.50%	98.03%	97.28%	0.9370	0.9368	0.9613
	400×	98.79%	98.39%	98.59%	98.09%	0.9563	0.9563	0.9721
	Average testing performance	97.82%	97.29%	97.56%	96.65%	0.9222	0.9221	0.9525

Moreover, the proposed method exhibited significant improvements in other evaluation metrics such as the MCC, Cohen’s kappa, and CSI. Specifically, the MCC increased from 0.8900 at 40× magnification to 0.9563 at 400×, indicating a strong correlation between the predicted and actual classes. Cohen’s kappa showed a similar trend, improving from 0.8899 to 0.9563, which reflects a substantial agreement beyond chance. The CSI also increased from 0.9336 to 0.9721 across the magnifications, demonstrating a higher proportion of correctly identified positive cases relative to all relevant instances. The average MCC, Cohen’s kappa, and CSI values were 0.9222, 0.9221, and 0.9525, respectively. These enhancements across multiple metrics highlighted the robustness and reliability of the proposed method for accurately classifying images at various magnifications.

Overall, the high accuracy and significant improvements in the MCC, Cohen’s kappa, and CSI underscore the potential of the proposed method for applications that require precise classification across varying magnifications. These results demonstrate not only the ability of the method to generalize effectively but also its robustness and reliability in producing consistent and high-quality classifications. The advancements showcased by the proposed method highlight its potential for enhancing the capabilities of ViT models, addressing the challenges posed by complex and diverse datasets, and potentially setting new benchmarks in the field of medical image classification.

#### Scenario 5: Increasing the outcomes by ensemble with the VGGIN model

Scenario 5 focused on using ensemble learning techniques to enhance the performance of the proposed model by combining it with the VGGIN model. The analysis in Table 7 indicates that the ensemble model that combines the proposed method with the VGGIN model achieved significant improvements over the model without VGGIN. Specifically, the accuracy increased from 95.94% to 97.13%, representing an improvement of 1.19%. The precision improved by 0.70% (from 97.56% to 98.26%), the recall increased by 1.04% (from 96.55% to 97.59%), and the F1-score increased by 0.87% (from 97.05% to 97.92%). In addition, the MCC improved from 0.9055 to 0.9331, indicating a stronger correlation between the predicted and actual classes. Cohen’s kappa increased from 0.9054 to 0.9331, reflecting a higher agreement beyond chance, and the CSI improved from 0.9428 to 0.9593, demonstrating a greater proportion of correctly identified positive cases.

**Table 7:** Results of the combination with the VGGIN model

Method	Precision	Recall	F1-score	Accuracy	MCC	Cohen’s kappa	CSI
Ensemble with VGGIN	98.26%	97.59%	97.92%	97.13%	0.9331	0.9331	0.9593
Without VGGIN	97.56%	96.55%	97.05%	95.94%	0.9055	0.9054	0.9428

The significant performance improvement achieved by the ensemble model highlights the effectiveness of combining diverse models with complementary strengths. By integrating the proposed model with the VGGIN model, the ensemble benefits from the diverse representations and decision boundaries learned by each model. Furthermore, the ensemble approach mitigates the risk of overfitting and enhances generalization by leveraging the collective knowledge of multiple models.

## Results and Discussion

**Fig. 11:**
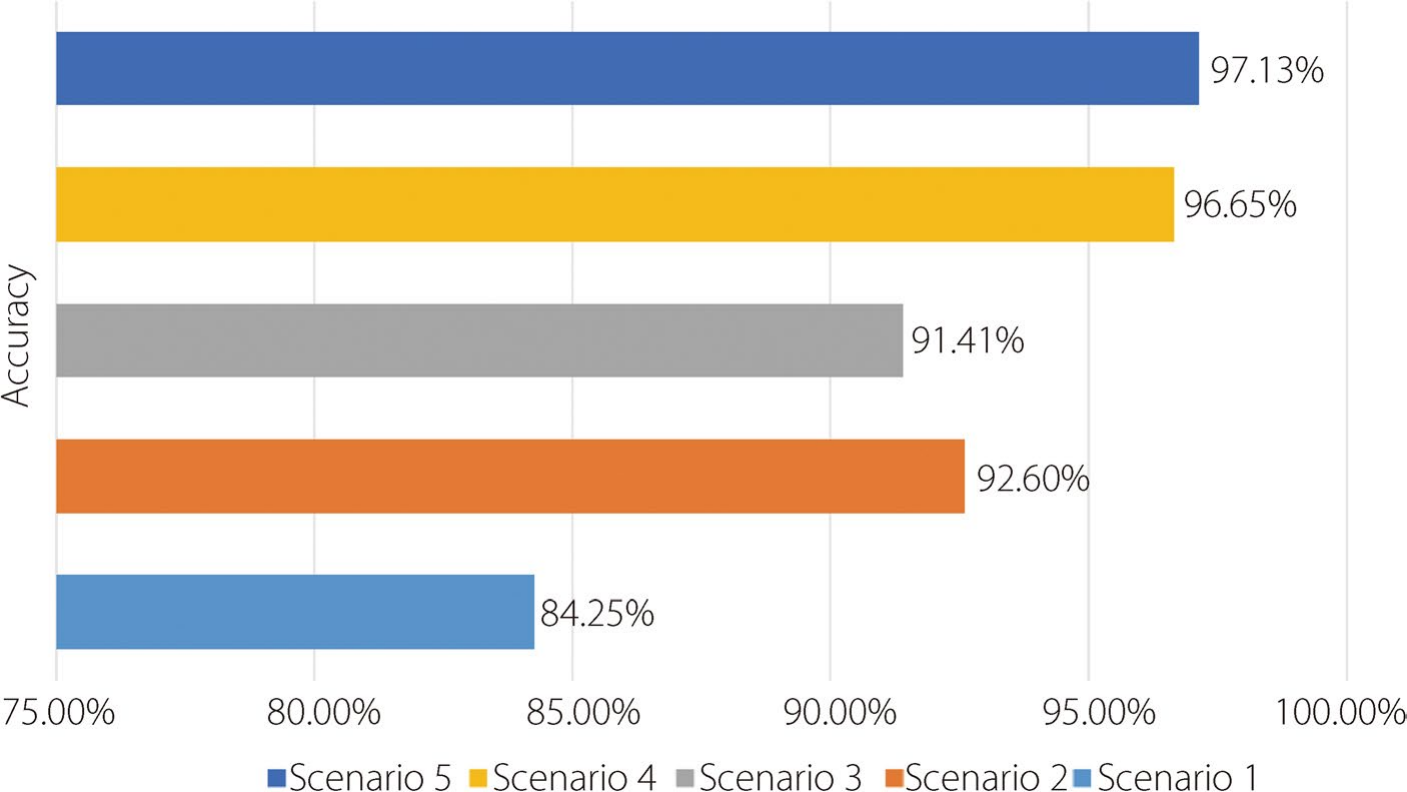
Comparison of results across five scenarios

Figure 11 summarizes the accuracies achieved in five distinct scenarios using different methodologies and techniques in ViT. In Scenario 1, the effect of PCA on data preprocessing was evaluated, and the accuracy reached 84.25%. In Scenario 2, various data augmentation techniques were explored, and the accuracy increased significantly to 92.60%. Scenario 3 introduced a novel attention mechanism, MLLKSA, which resulted in an accuracy of 91.41%. In Scenario 4, a comprehensive combination of preprocessing, feature extraction, and magnification techniques led to a significant accuracy increase of 96.65%. Finally, Scenario 5 illustrated the effectiveness of ensemble learning by combining the proposed model with the VGGIN model, achieving the highest accuracy of 97.13%.

In summary, MLLKSA is designed to capture a wide range of spatial information, from local textures to broader spatial patterns. By integrating large kernel layers, this mechanism enhances the ability to capture spatial relationships across different scales, contributing to more comprehensive feature extraction and improved model performance. In addition, both the PCA and data augmentation techniques significantly enhance the model performance in machine learning tasks. PCA aids in reducing the dimensionality of the input data and capturing its underlying structure, whereas data augmentation enriches the training set with diverse variations. Integrating these techniques into the model training pipeline can lead to more efficient, robust, and generalizable machine -learning models.

In conclusion, the comparison across the five scenarios underscores the effectiveness of various methodologies and techniques in enhancing the performance and interpretability of ViT. By systematically exploring different preprocessing, augmentation, attention mechanisms, and ensemble learning approaches, the model achieved significant improvements in accuracy and demonstrated the potential of ViT models for tackling complex classification tasks.

A comparison of the proposed model with other state-of-the-art methods using the BreakHis dataset provides valuable insights into its performance and competitiveness. Table 8 presents a comprehensive overview of various models and their corresponding accuracies. In this context, the proposed model indicated that MLLKSA and PCA achieved accuracies of 96.65% and 97.13%, respectively, demonstrating their effectiveness and competitive performance compared to other state-of-the-art methods. This comparison underscores the significance of novel attention mechanisms and preprocessing techniques for advancing breast cancer classification and the potential of the proposed model in clinical applications.

**Table 8:** Comparison of the proposed model with other state-of-the-art models on the BreakHis dataset

**Reference**	**Method**	**Accuracy**
[15]	VGG-19	80.00%
[16]	VGGIN-Net	96.15%
[17]	DenseNet and XGBoost	94.12%
[18]	Inception and SEP	83.73%
[19]	AlexNet and VGG16	91.05%
[20]	ResHist	92.52%
[21]	FE-BkCapsNet	93.70%
[22]	DenseNet and SENet	87.90%
[23]	ResNeXt	91.09%
[24]	DenseNet201 and XGBoost	91.95%
[25]	DarkNet53	87.17%
[29]	MaxViT	91.57%
[28]	DecT	93.02%
**Ours**	**MLLKSA and PCA**	**96.65%**
		**97.13%**

## Conclusions

This study explored the efficacy of different methodologies and techniques for enhancing the performance of ViT models. Combining several methods, such as the MLLKSA mechanism, ensemble learning with the VGGIN model, data preprocessing with PCA, and various data augmentation techniques, led to significant improvements in classification accuracy. Thus, the average research accuracies of 96.65% and 97.13% on the BreakHis dataset were effective for breast cancer classification.

Despite these promising results, our study has some limitations that must be acknowledged. The computational complexity of our methods, particularly the integration of sophisticated attention mechanisms and use of ensemble models, poses significant challenges. These approaches require substantial computational resources, which can limit their applicability in resource-constrained environments. Moreover, while strides have been made to improve the interpretability of ViT models through techniques such as SHAP, LIME, and Grad-CAM, the underlying mechanisms of these models remain complex and may present challenges in clinical settings, where interpretability and trust are paramount. Furthermore, our study primarily focused on a single dataset, which, while robust, does not encompass the full diversity of histopathological variations observed in breast cancer globally. Future studies should aim to validate these findings across more varied datasets to enhance the generalizability of the proposed methods.

Given these limitations, future research should focus on optimizing the computational efficiency of these models, perhaps by simplifying the architecture without compromising the performance. In addition, efforts should be made to enhance the interpretability of these models to make them more accessible and trustworthy for clinical practitioners. Expanding the application of these models to a broader array of datasets would also be a critical next step, helping to ensure that the solutions developed are robust and universally applicable.

In summary, this study highlights the potential of ViT models and fine-tuning techniques for advancing image classification tasks. It employs attention mechanisms, preprocessing techniques, and ensemble learning to achieve surprising accuracy. Although limitations exist, ongoing research and innovation hold promise for further enhancing the efficacy and applicability of ViT models in clinical practice and beyond.

## Data Availability

The data supporting this study’s findings is available at https://web.inf.ufpr.br/vri/databases/breast-cancer-histopathological-database-breakhis/.

## Abbreviations

ViT: Vision Transformer
PCA: Principal component analysis
SHAP: SHapley Additive exPlanations
LIME: Local Interpretable Model-agnostic Explanations
Grad-CAM: Gradient-weighted Class Activation Mapping
SVM: Support vector machine
MLLKSA: Multi-head locality large kernel self-attention
CNN: Convolutional neural network
ROC: Receiver operating characteristic
AUC: Area under the curve
MCC: Matthews correlation coefficient
CSI: Critical success index
AI: Artificial intelligence
DCNN: Deep CNN
DecT: Deconv-Transformer
ROC: Receiver operating characteristic
TP: True positives
TN: True negatives
FP: False positives
FN: False negatives
